# Supplementary material for: Evolution of SARS-CoV-2 spike trimers towards optimized heparan sulfate cross-linking and inter-chain mobility
Source: Sci Rep. 2024 Dec 31;14:32174. doi: 10.1038/s41598-024-84276-5 (PMC11688500; doi:10.1038/s41598-024-84276-5)
Supplement: Supplementary file 1 — Supplementary Material 1 [file 41598_2024_84276_MOESM1_ESM.pdf]

Supplementary data for

Evolution of SARS-CoV-2 spike trimers towards optimized heparan sulfate cross-linking and inter-chain mobility

Jurij Froese[1], Marco Mandalari [2,3], Monica Civera[3], Stefano Elli[2], Isabel Pagani[4], Elisa Vicenzi[4], Itzel Garcia-Monge[6], Daniele Di Iorio[1], Saskia Frank[1], Antonella Bisio[2], Dominik Lenhart[7], Rudolf Gruber[7], Edwin A. Yates[5], Ralf P. Richter[6], Marco Guerrini[2], Seraphine V. Wegner[1], Kay Grobe[1]

Institutions:

1. Institute of Physiological Chemistry and Pathobiochemistry, University of Münster, Waldeyerstrasse 15, D-48149 Münster, Germany.
2. Istituto di Ricerche Chimiche e Biochimiche "G. Ronzoni", via Giuseppe Colombo 81, 20133 Milano, Italy.
3. Università degli Studi di Milano, Dipartimento di Chimica, Via Golgi 19 20133 Milano, Italy.
4. Viral Pathogenesis and Biosafety Unit, Division of Immunology, Transplantation and Infectious Diseases, IRCCS San Raffaele Scientific Institute, Milan, Italy.
5. Department of Biochemistry, Systems and Cell Biology, Institute of Molecular, Integrative and Systems Biology, University of Liverpool, Liverpool L69 7ZB, UK.
6. School of Biomedical Sciences, Faculty of Biological Sciences, School of Physics and Astronomy, Faculty of Engineering and Physical Sciences, Astbury Centre for Structural Molecular Biology, and Bragg Centre for Materials Research, University of Leeds, LS2 9JT Leeds, UK.
7. Bene PharmaChem GmbH & Co.KG, Geretsried, Germany.

This pdf file includes:
Supplementary figures 1-6
Supplementary tables 1-5

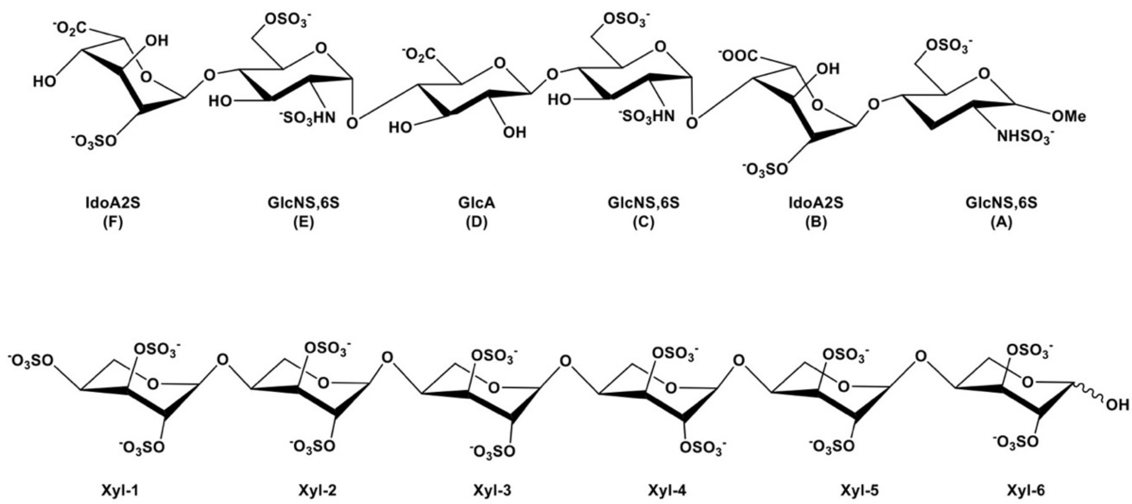

Supplementary Figure 1: Structure of IAGAIA (top) and PPS (bottom) ligands used for docking experiments with RDB protein variants.

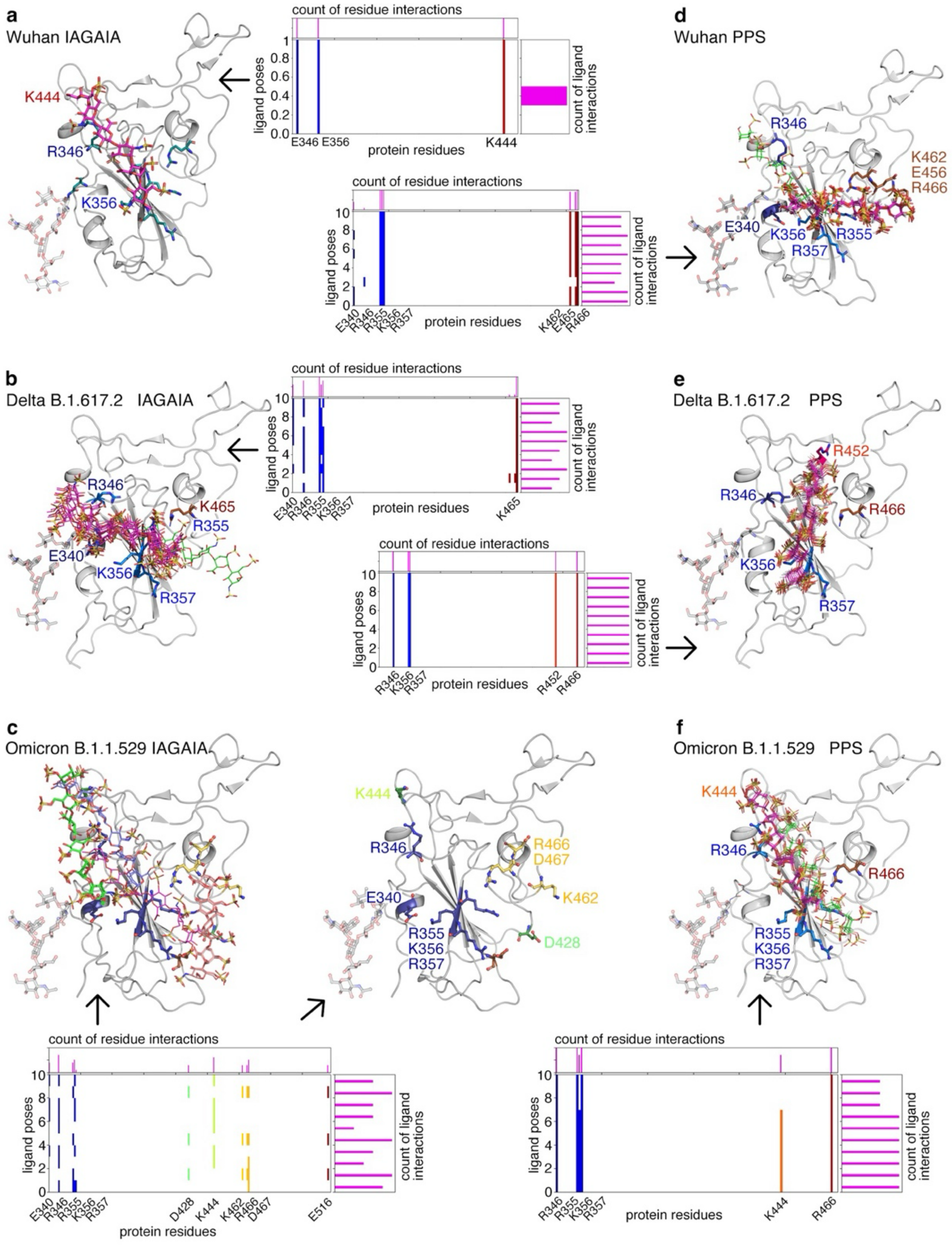

Supplementary Figure 2: Analysis of the docking pose interactions of IAGAIA (a,c,e) and PPS (b,d,f) in the Wuhan, Delta B.1.617.2, and Omicron B.1.1.529 RBD models. Key residues are labelled in the figure and for each docking pose the charged residues making interactions with the ligand are shown in the graph below. Moving from IAGAIA to PPS the ligand is prone to exhibit a smaller number of binding modes due to the more focused interaction with basic residues exposed in the basic channel located in the binding site. The images were generated in Schroedinger Maestro 2023-1 and the figure assembled using Photoshop 2023 under a university licence.

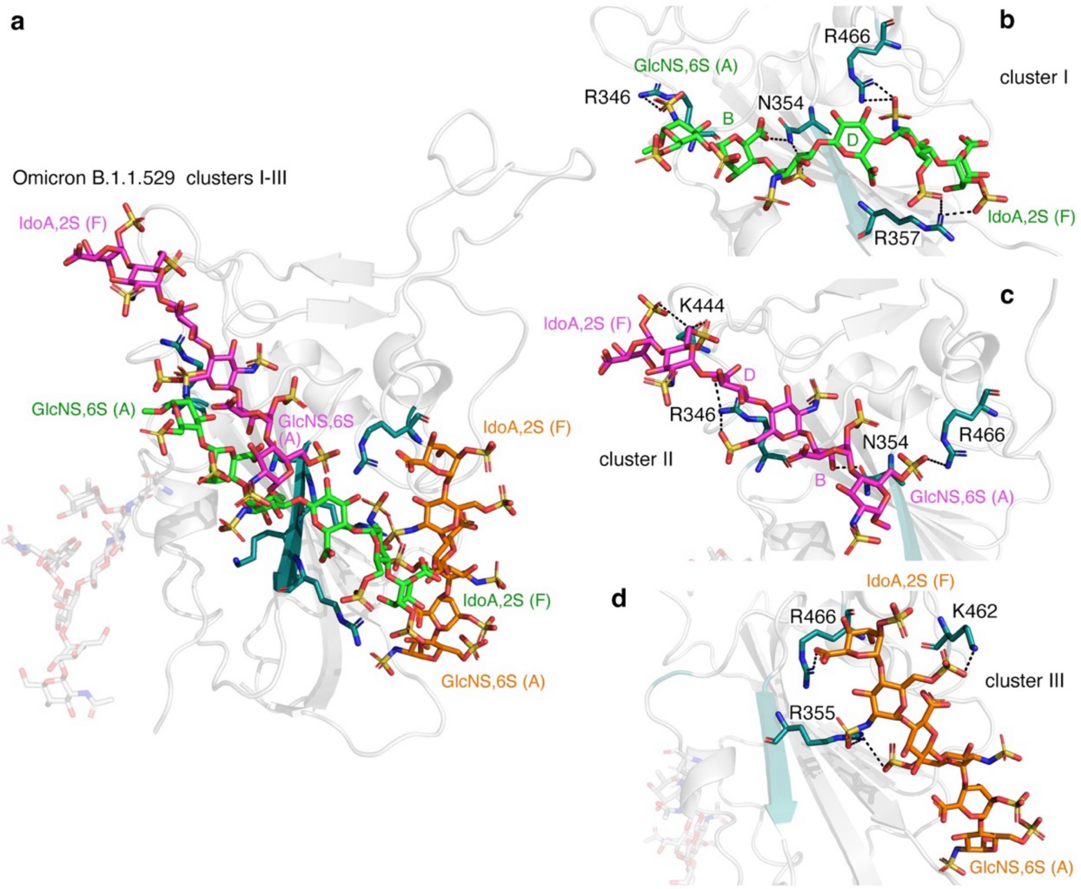

Supplementary Figure 3: a) IAGAIA and Omicron B.1.1.529 RBD cluster I (green), II (magenta), and III (orange). **b-d)** Best se of cluster I, II and III. The RBD region is represented by a grey cartoon, while IAGAIA represented red, blue and yellow sticks indicating oxygen, nitrogen and sulfur atoms, respectively. The key interacting residues of the pocket are labelled and depicted with similar color codes (deep teal for carbon, red for oxygen and blue for nitrogen), while H bond and salt bridges are represented as black dashed lines. The images were generated in Schroedinger Maestro 2023-1 and the figure assembled using Photoshop 2023 under a university licence.

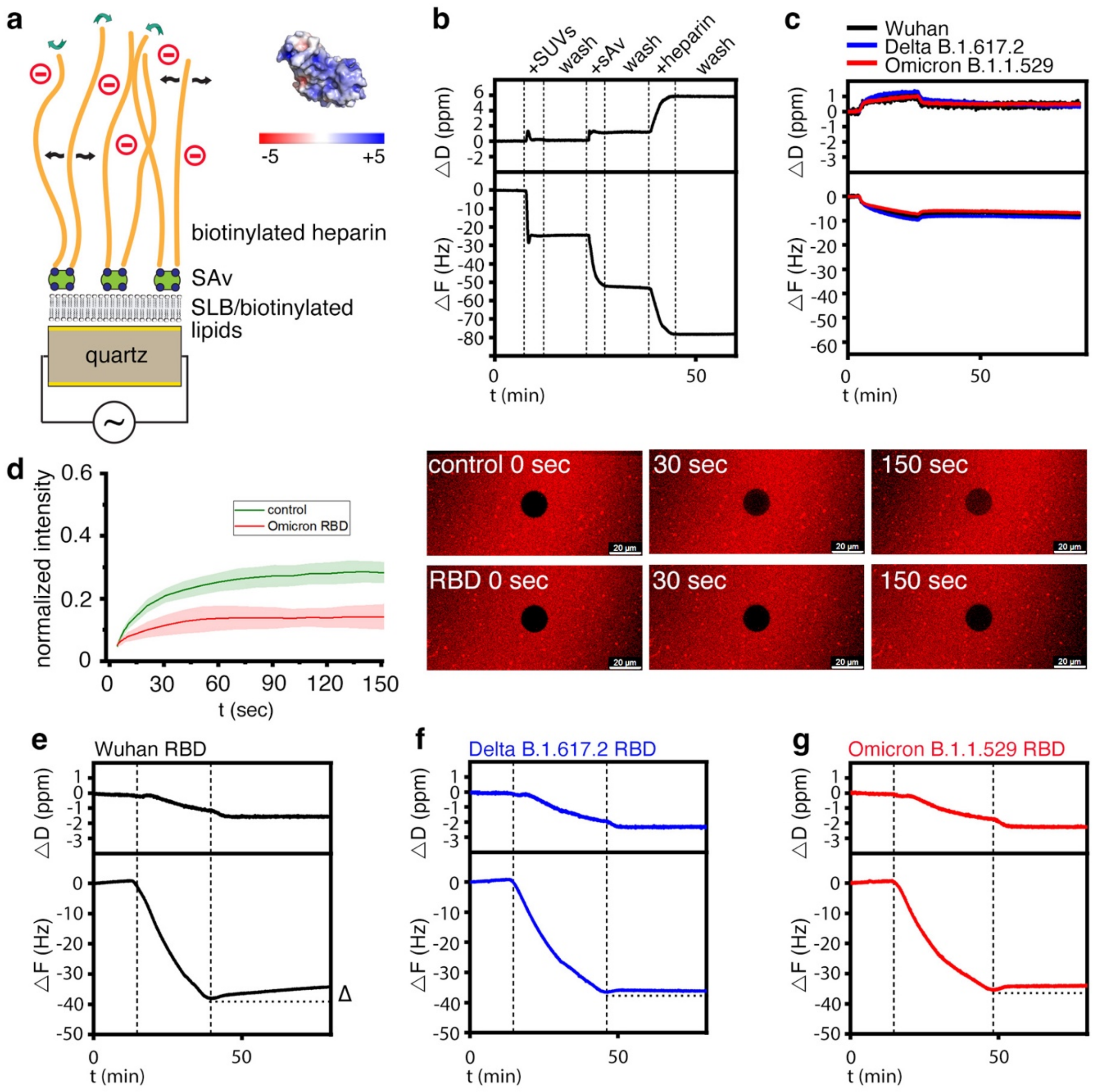

Supplementary Figure 4: Quartz crystal microbalance with dissipation monitoring (QCM-D). a) The core of the QCM technology is a gold-coated oscillating quartz crystal sensor disk with a resonance frequency related to the mass of the disk. This allows the real-time detection of nanoscale mass changes on the sensor surface by monitoring changes in the resonance frequency (ΔF). Interaction surfaces containing biotinylated (blue) heparin (orange) linked via steptavidin (green) to fluid supported lipid bilayers (SLBs, black) were generated as a proxy for cell-surface-bound HS. Like cell surface HS, SLB-linked highly negatively charged heparin can freely rotate (green curved arrows) and potentially move laterally (black arrows) on the sensor surface. Adsorption of positively charged molecules on the heparin-covered surface decreases F, and mass loss during washing increases F. QCM-D measures an additional parameter, the change in energy dissipation D, which is particularly useful for studying viscoelastic properties of the layer. An increased ΔD during protein binding to the functionalized surface correlates with a softer layer, and a decreased ΔD would indicate layer stiffening, for example by cross-linking of heparin chains by the bound molecules. **b)** Preparation and validation of a heparin/HS cell surface matrix model for QCM-D. Representative QCM-D data displaying the observed frequency (ΔF) and dissipation (ΔD)

shifts during assembly of the SLB, the SAv monolayer and the film of end-attached heparin on the sensor's silica surface. Start and duration of sample incubations are indicated by dashed vertical lines and a label on top of the graph. At all other times, the surface was exposed to wash buffer (10 mM Tris pH 7.4, 100 mM NaCl). The formation of the heparin/HS model matrix on the QCM sensor was always followed in real-time prior to the protein incubation assays shown in Figures 3-5 and Supplementary Figures 2-4 to validate the surface functionalization. **c)** A control addition of RBDs to a surface without terminally attached heparin shows much smaller responses than on heparin, confirming that RBD binding to heparin is largely specific. **d)** The Omicron RBD reduced the capacity of fluorescently labeled, streptavidin-coupled heparin to move laterally on supported lipid bilayers. Right: Representative fluorescence micrographs displaying FRAP results on heparin coated surfaces. Shown are bleach areas at 0 s, 30 s and 150 s after the bleaching, as indicated. The fluorescence is partly recovered for a bare HS layer after 150 s, indicating rapid diffusion of the SAv-anchored HS (SAv-atto565 was here used as mobility tracer), RBD/heparin layers retain a substantially higher amount of bleaching, consistent with reduced diffusivity of HS due to RBD-mediated cross-linking. Note that because the RBD is small and can move quickly between the surface-bound chains, the cross-links are transient and therefore the inhibition of lateral heparin diffusion is less pronounced than would be expected from permanent cross-linking. **e-g)** Representative QCM-D data (shown analogous to Fig. 3a) for model matrix formation with heparin and subsequent RBD interaction to better discriminate variant unbinding responses. The start and duration of sample incubations are indicated by dashed vertical lines and a label at the top of the graph. At all other times, the surface was exposed to wash buffer. Note the overall similarity in RBD binding to heparin, but the different stability of the interaction when washed in buffer (the dotted horizontal line represents $-\Delta F$ at the start of the buffer wash). We also note that the Wuhan, Delta and Omicron RBDs are of similar size, allowing for direct comparison in this assay. S-trimer measurements shown elsewhere in this paper should not be directly compared due to possible variations in close/open conformations and increased size, which are likely to affect binding and unbinding rates. However, S-trimer binding between the variants is comparable as shown in this paper.

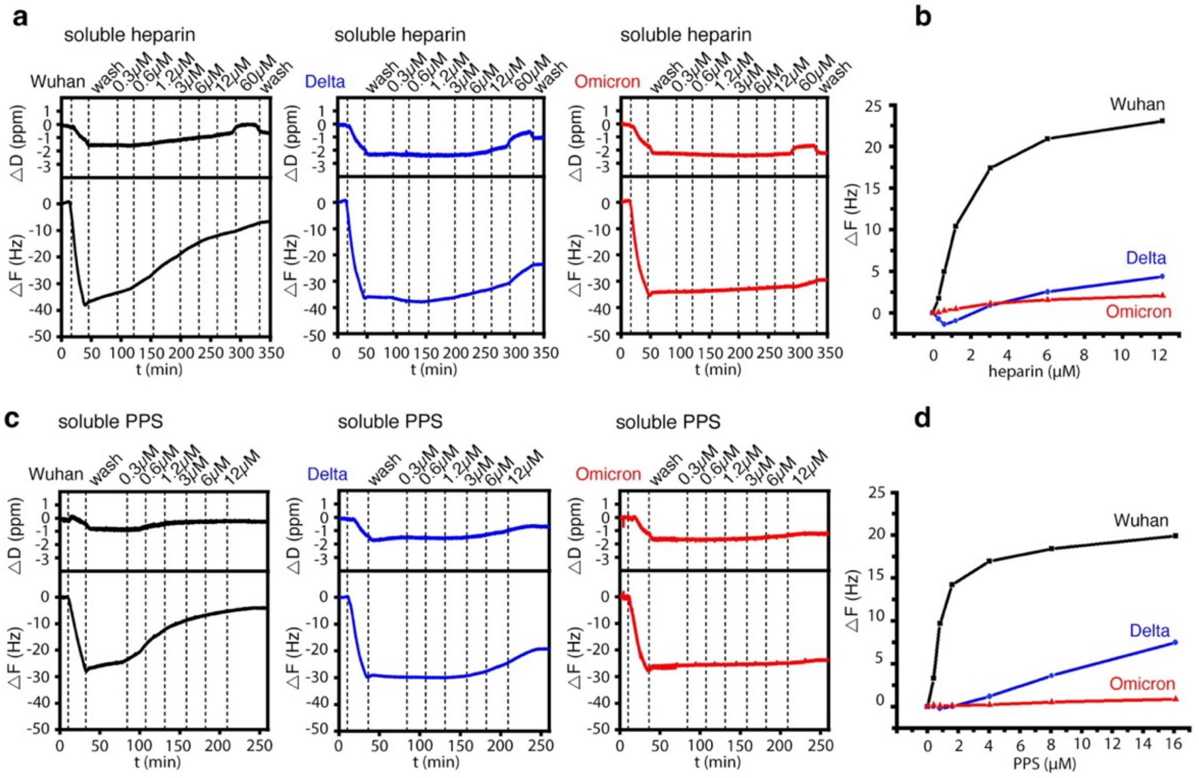

Supplementary Figure 5: Soluble heparin and PPS increase the dissociation of the Wuhan and Delta B.1.617.2 RBDs, but not that of the Omicron B.1.1.529 RBD. a) Individual graphs of data shown in Figure 4a. The dissociation of the Wuhan S-protein RBD from the sensor surface increases with increasing heparin concentration in the washing buffer, and the Delta S-protein RBD also dissociates at higher heparin concentrations. The Omicron RBD does not dissociate from the heparin-functionalized sensor surface. **b)** Relative heparin elution strength for the Wuhan, Delta and Omicron RBDs from the sensor surface. The starting point of the curves is represented by the -ΔF value at the end of the wash step. **c)** As shown in Figure 4b, increasing the negative charge of soluble PPS in the wash buffer increases the dissociation of the Wuhan and Delta RBDs, but not of the Omicron RBD. **d)** Relative PPS elution strength for the Wuhan, Delta and Omicron RBDs from the sensor surface.

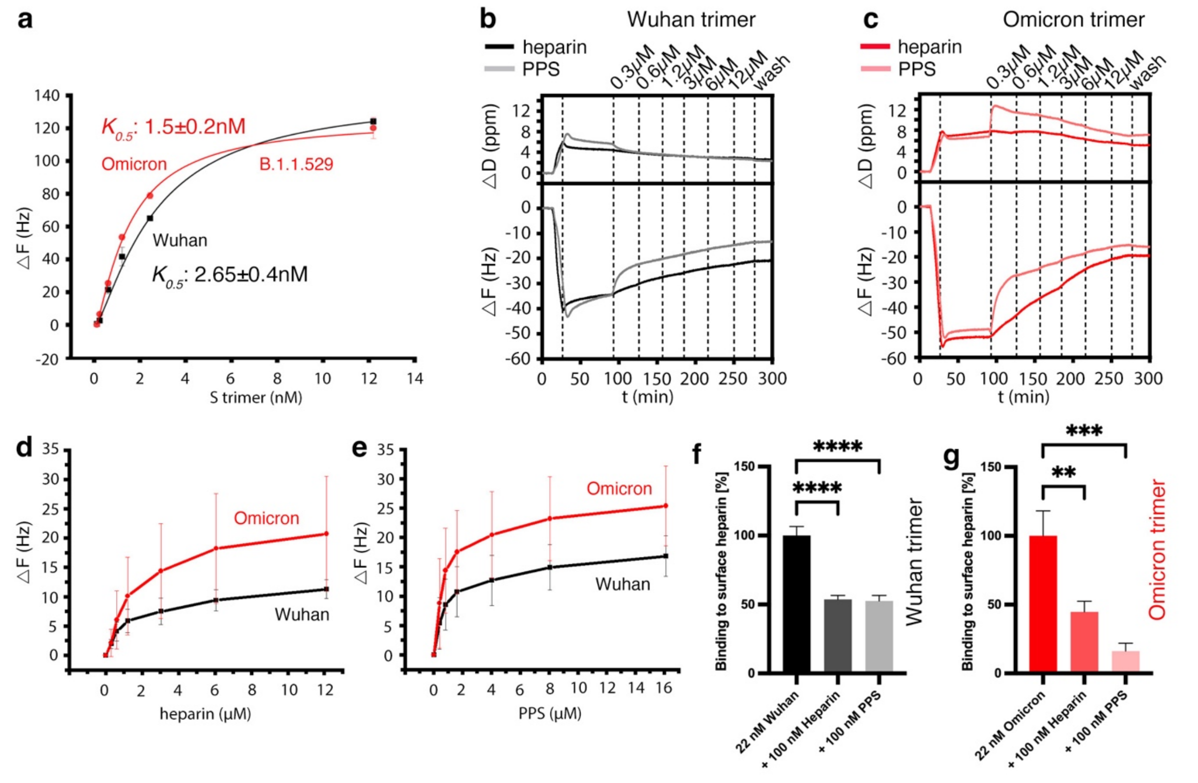

Supplementary Figure 6: Strongly increased Omicron S-trimer switching to the soluble PPS acceptor indicates charge-driven direct switching of the protein. a) Hill fit of QCM-D binding data obtained at different S-trimer concentrations. The concentration of half-maximal binding $K_{0.5}$ of the Wuhan S-trimer exceeded that of the Omicron B.1.1.529 variant by a factor of approximately 2. **b)** Low amounts of soluble heparin and PPS readily desorb Wuhan S-trimers from the heparin-functionalized sensor surface, with PPS being more effective (note the steep slope immediately after the addition of soluble PPS, gray line, asterisk). **c)** The same low amounts of soluble heparin and PPS desorb Omicron S-trimers much more strongly from the heparin-functionalized sensor surface, with PPS again being most effective. Note that the striking increase in trimeric Omicron S-protein desorption was accompanied by a concomitant increase in *D*. One possibility to explain this result is a (non-specific) association of PPS with the sensor surface. However, this possibility is unlikely because of the strong repulsion between the equally charged PPS and the immobilized heparin. It is also unlikely because higher amounts of PPS in the wash buffer do not further increase *D*. Therefore, we suggest that the observed increase in *D* may be due to the rapid de-crosslinking of the heparin film by the trimeric S-protein as a prerequisite for their subsequent switch to the more negatively charged PPS and effective release of the protein/PPS complex into the solution phase. **d)** Relative heparin elution strength for the Wuhan and Omicron S-protein trimers from the sensor surface. The starting point of the curves is represented by the -ΔF value at the end of the wash step. **e)** Relative PPS elution strength for the Wuhan and Omicron S-protein trimers from the sensor surface. **f)** Relative inhibition of Wuhan S-protein trimer association to the sensor surface upon protein preincubation with or without 100nM soluble heparin or PPS. One-way ANOVA, Dunnet's multiple comparison test, n=3. ****: $p<0.0001$. **g)** Relative inhibition of Omicron S-protein trimer association to the sensor surface in the absence or presence of 100nM heparin or PPS. One-way ANOVA, Dunnet's multiple comparison test, n=1 for the Omicron control, n=2 for the PPS-treated protein and n=3 for the heparin-treated probe.

Supplementary Table 1: IAGAIA re-scored MMGBSA values and docking scores for the poses obtained for the Wuhan strain RBD and Delta and Omicron RBDs.

IAGAIA

RDB	Pose	Docking Score (kcal/mol)	MMGBSA (kcal/mol)
WT (6M0J)	1	-1.51	-17.67
Delta (7V8B)	1	-3.95	-35.27
	2	-3.58	-34.40
	3	-2.76	-28.71
	4	-2.72	-31.74
	5	-2.52	-27.66
	6	-2.52	-34.80
	7	-1.91	-18.26
	8	-1.74	-18.41
	9	-1.67	-15.61
	10	-1.03	-13.79
Omicron (7WBP)	1	-5.09	-37.40
	2	-4.92	-35.35
	3	-4.80	-31.02
	4	-4.63	-42.44
	5	-4.53	-33.34
	6	-4.41	-35.70
	7	-4.08	-31.69
	8	-3.97	-34.41
	9	-3.77	-24.99
	10	-3.77	-29.84

Supplementary Table 2: PPS re-scored MMGBSA values and docking scores for the poses obtained for the Wuhan strain RBD and the Delta and Omicron RBDs .

PPS

RDB	Pose	Docking Score (kcal/mol)	MMGBSA (kcal/mol)
WT (6M0J)	1	-4.74	-19.21
	2	-4.22	-26.41
	3	-4.15	-24.43
	4	-3.85	-25.16
	5	-3.85	-27.08
	6	-3.82	-24.91
	7	-3.78	-20.75
	8	-3.74	-27.33
	9	-3.72	-25.31
	10	-3.72	-21.72
Delta (7V8B)	1	-5.13	-29.08
	2	-4.85	-24.63
	3	-4.70	-23.72
	4	-4.50	-31.08
	5	-4.49	-27.32
	6	-4.47	-15.36
	7	-4.47	-28.92
	8	-4.29	-28.94
	9	-4.24	-30.62
	10	-4.11	-31.04
Omicron (7WBP)	1	-5.76	-47.28
	2	-5.55	-44.35
	3	-5.19	-42.98
	4	-5.16	-46.56
	5	-5.09	-59.84
	6	-5.07	-42.28
	7	-5.02	-51.16
	8	-3.67	-35.94
	9	-3.22	-32.38
	10	-3.15	-38.95

Supplementary Table 3: Salt bridges (SB) and hydrogen bonds (HB) established in IAGAIA and PPS docking with the S1-RBD of the Wuhan strain RBD and the Delta and Omicron variants. The pair of atoms on which the distance is calculated are in bold.

		Protein residue	Ligand residue	Distance
IAGAIA	**Wuhan**	K444-**N**H_3^+	IdoA2S(F)-**C**OO^-	4.2Å (SB)
		K356-**N**H_3^+	GlcNS,6S(A)-N-**S**O_3^-	3.8Å (SB)
		R346-NH**C**NH_2^+	GlcNS,6S(E)-3-**O**H	3.9Å (HB)
	**Delta**	R357-NH**C**NH_2^+	IdoA2S(F)-2-O-**S**O_3^-	3.5Å (SB)
		R357 C_α**N**H	IdoA2S(F)-3-**O**H	3.1Å (HB)
		R346-NH**C**NH_2^+	IdoA2S(B)-**C**OO^-	4.4Å (SB)
		R346-NH**C**NH_2^+	GlcNS,6S(C)-6-O-**S**O_3^-	4.0 Å (SB)
		N354-C**N**H_2	GlcNS,6S-N-**S**O_3^- (E)	4.6 Å (SB)
		N354-C**N**H_2	GlcNS,6S-6-O-**S**O_3^- (C)	3.6 Å (SB)
	**Omicron Cluster I**	R346-C_α**N**H	GlcNS,6S-N-**S**O_3^-	4.7Å (HB)
		R357-NH**C**NH_2^+	IdoA2S-2-O-**S**O_3^-	5.0Å (SB)
		N354-C_α**N**H_2	IdoA2S(B)-**C**OO^-	4.5Å (HB)
		N354-C_α**N**H_2	GlcNS,6S(C)-6-O-**S**O_3^-	3.4Å (HB)
		R466-HN**C**NH_2^+	GlcNS,6S(E)-N-**S**O_3^-	4.0 Å (SB)
		R357- HN**C**NH_2^+	GlcNS,6S(E)-6-O-**S**O_3^-	4.8 Å (HB)
	**Omicron Cluster II**	R466-HN**C**NH_2^+	GlcNS,6S(A)-4-O-**S**O_3^-	4.0 Å (SB)
		K444-**N**H_3^+	IdoA2S(F)-O-2-**S**O_3^-	4.0Å (SB)
		K444-**N**H_3^+	GlcNS,6S(E)-6-O-**S**O_3^-	3.2Å (SB)
		R346-HN**C**NH_2^+	GlcA(D)-**C**OO^-	3.3Å (SB)
		R346-HN**C**NH_2^+	GlcNS,6S(C)-6-O-**S**O_3^-	3.8 Å (SB)
	**Omicron Cluster III**	R355-HN**C**NH_2^+	GlcNS,6S(E)-N-**S**O_3^-	4.7Å (HB)
		R355-HN**C**NH_2^+	GlcNS6S-6-O-**S**O_3^-	4.0 Å (SB)
		R466-HN**C**NH_2^+	IdoA2S(F)-**C**OO^-	4.9Å (HB)
		R466-C_α**N**H	IdoA2S(F)-3-**O**H	2.9Å (HB)
		K462-**N**H_3^+	GlcNS,6S(E)-6-O-**S**O_3^-	3.5Å (SB)

PPS	**Wuhan Cluster I**	K462-**N**H_3^+	Xyl-1– 2-O-**S**O_3^-	3.7Å (SB)
		K462-**N**H_3^+	Xyl-1– 4-O-**S**O_3^-	3.6Å (SB)
		N354-C**N**H_2	Xyl-6 – 3-O-**S**O_3^-	5.0 Å (SB)
		K356-**N**H_3^+	Xyl-6 – 3-O-**S**O_3^-	3.5 Å (HB)
		R466-HN**C**NH_2^+	Xyl-3 – 3-O-**S**O_3^-	3.8 Å (SB)
		R466-HN**C**NH_2^+	Xyl-4 – 2-O-**S**O_3^-	4.2Å (SB)
		R355- HN**C**NH_2^+	Xyl-2 – 2-**S**O_3^-	4.8Å (SB)
		K356-**N**H_3^+	Xyl-5 – 3-**S**O_3^-	4.8Å (SB)
		R357- HN**C**NH_2^+	Xyl-4 – 2-**S**O_3^-	4.1Å (HB)
		E340-**C**NH_2	Xyl-6 – 4-**O**H	4.0Å (HB)
	**Wuhan Cluster II**	R357-C_α**N**H	Xyl-1 – 2-O-**S**O_3^-	3.6Å (HB)
		R466- HN**C**NH_2^+	Xyl-1 – 4-O-**S**O_3^-	4.6Å (SB)
		R355-**C**NH_2	Xyl-1 – 2-O-**S**O_3^-	3.8Å (HB)
		K356-**N**H_3^+	Xyl-1 – 4-O-**S**O_3^-	6.4Å (SB)
		K356-**N**H_3^+	Xyl-2 – 2-O-**S**O_3^-	3.8Å (SB)
		K356-**N**H_3^+	Xyl-2 – 3-O-**S**O_3^-	5.7Å (SB)
		R346-C_α**N**H	Xyl-4 – 2-O-**S**O_3^-	4.2 Å (HB)
		R346-NH**C**NH_2^+	Xyl-6 – 3-O-**S**O_3^-	

			4.1 Å<br>(SB/HB)
**Delta**	R452-NH**C**NH$_2^+$<br>R346-NH**C**NH$_2^+$<br>K356-**N**H$_3^+$<br>K356-**N**H$_3^+$<br>K356-**N**H$_3^+$<br>N354-C$_\alpha$**N**H<br>N354-C$_\alpha$**N**H<br>R466-HN**C**NH$_2^+$	Xyl-1 – 3-O-**S**O$_3^-$<br>Xyl-4 – 3-O-**S**O$_3^-$<br>Xyl-1 – 4-O-**S**O$_3^-$<br>Xyl-2 – 2-O-**S**O$_3^-$<br>Xyl-2 – 2-O-**S**O$_3^-$<br>Xyl-4 – 2-O-**S**O$_3^-$<br>Xyl-4 – 2-O-**S**O$_3^-$<br>Xyl-4 – 2-O-**S**O$_3^-$	5.1Å (SB/HB)<br>4.1Å (SB)<br>4.5Å (SB)<br>5.9Å (SB)<br>6.0Å (SB)<br>3.4Å (HB)<br>4.0Å (HB)<br>4.1Å (SB/HB)
**Omicron Cluster I**	K444-**N**H$_3^+$<br>K444-**N**H$_3^+$<br>R346-NH**C**NH$_2^+$<br>R346-NH**C**NH$_2^+$<br>R346-NH**C**NH$_2^+$<br>R357-C$_\alpha$**N**H<br>R466- HN**C**NH$_2^+$<br>R357-C$_\alpha$**N**H	Xyl-1 – 2-O-**S**O$_3^-$<br>Xyl-1 – 3-O-**S**O$_3^-$<br>Xyl-1 – 3-O-**S**O$_3^-$<br>Xyl-1 – 3-O-**S**O$_3^-$<br>Xyl-4 – 2-O-**S**O$_3^-$<br>Xyl-6 – 2-O-**S**O$_3^-$<br>Xyl-6 – 2-O-**S**O$_3^-$<br>Xyl-6 – 3-O-**S**O$_3^-$	3.5Å (SB)<br>4.0Å (SB)<br>4.7Å (SB)<br>4.7Å (SB)<br>5.3Å (SB)<br>4.2Å (HB)<br>4.6Å (SB)<br>3.8Å (HB)
**Omicron Cluster II**	N450 C$_\alpha$**O**NH$_2$<br>R355-C$_\alpha$**N**H<br>R466- HN**C**NH$_2^+$<br>R466- HN**C**NH$_2^+$<br>R357- HN**C**NH$_2^+$	Xyl-6 – 4-**O**H<br>Xyl-3 – 3-O-**S**O$_3^-$<br>Xyl-3 – 3-O-**S**O$_3^-$<br>Xyl-3 – 3-O-**S**O$_3^-$<br>Xyl-1 – 4-O-**S**O$_3^-$	2.7Å (HB)<br>4.7Å (HB)<br>5.3Å (SB)<br>4.5Å (SB/HB)<br>5.0Å (SB/HB)

Supplementary Table 4: Statistical analysis of data presented in Figure 6

**Fig. 6a**	Wuhan D614G	Mean±SD: 60.5±5		**n=6**
	+ heparin	Mean±SD: 23.5±2.6	p<0.0001	n=6
	Omicron BA.1	Mean±SD: 55.2±5		n=6
	+ heparin	Mean±SD: 28.8±4.5	p<0.0001	n=6
**Fig. 6b**	Wuhan D614G	Mean±SD: 60.5±5		n=6
	+ heparin	Mean±SD: 34.5±2.7	p<0.0001	n=6
	Omicron BA.1	Mean±SD: 55.2±5		n=6
	+ heparin	Mean±SD: 32±2.9	p=0.0006	n=6
**Fig. 6c**	Wuhan D614G	Mean±SD: 60.5±5		n=6
	+ heparin	Mean±SD: 18.3±2.5	p<0.0001	n=6
	Omicron BA.1	Mean±SD: 55.2±5		n=6
	+ heparin	Mean±SD: 20.8±2	p<0.0001	n=6
**Fig. 6d**	Wuhan D614G	Mean±SD: 60.5±5		n=6
	+ PPS	Mean±SD: 23.7±2.2	p<0.0001	n=6
	Omicron BA.1	Mean±SD: 55.2±5		n=6
	+ PPS	Mean±SD: 12.7±2.2	p<0.0001, * p=0.0015	n=6
**Fig. 6e**	Wuhan D614G	Mean±SD: 60.5±5		n=6
	+ PPS	Mean±SD: 21.7±3.1	p<0.0001	n=6
	Omicron BA.1	Mean±SD: 55.2±5		n=6
	+ PPS	Mean±SD: 19.7±3	p=0.0003	n=6
**Fig. 6f**	Wuhan D614G	Mean±SD: 60.5±5		n=6
	+ PPS	Mean±SD: 13.8±3	p<0.0001	n=6
	Omicron BA.1	Mean±SD: 55.2±5		n=6
	+ PPS	Mean±SD: 5.3±2.6	p<0.0001, ** p<0.0381	n=6

Supplementary Table 5: PPS treatment efficacy quantification of Wuhan D416G versus Omicron BA.1

**Test conditions**	**Mean no PPS**	**Mean + PPS**	**Mean Diff**	**SE of Diff**	**fold reduction**
Cell pretreatment					
**Wuhan D416G vs Wuhan D416G + PPS**	60.50	23.67	36.83	2.136	**2.55**
**Omicron BA.1 vs Omicron BA.1 + PPS**	55.17	12.67	42.50	2.592	**4.35**
Virus pretreatment					
**Wuhan D416G vs Wuhan D416G + PPS**	60.50	21.67	38.83	1.701	**2.8**
**Omicron BA.1 vs Omicron BA.1 + PPS**	55.17	19.67	35.50	3.019	**2.8**
Cell + Virus pretreatment					
**Wuhan D416G vs Wuhan D416G + PPS**	60.50	13.83	46.67	2.603	**4.37**
**Omicron BA.1 vs Omicron BA.1 + PPS**	55.17	5.333	49.83	2.688	**10.34**
